# Opportunities for technologically driven dialogical health communication for participatory interventions: Perspectives from male peer navigators in rural South Africa

**DOI:** 10.1016/j.socscimed.2021.114539

**Published:** 2022-01

**Authors:** Andrew Gibbs, Dumsani Gumede, Manono Luthuli, Zakhele Xulu, Laura Washington, Yandisa Sikweyiya, Oluwafemi Adeagbo, Maryam Shahmanesh

**Affiliations:** a Gender and Health Research Unit, South African Medical Research Council, South Africa; b School of Nursing and Public Health, University of KwaZulu-Natal, South Africa; c African Health Research Institute (AHRI), Durban, South Africa; d Project Empower, Durban, South Africa; e School of Public Health, University of the Witwatersrand, Johannesburg, South Africa; f Department of Health Promotion, Education and Behaviour, University of South Carolina, Columbia, USA; g Department of Sociology, University of Johannesburg, South Africa; h Institute for Global Health, University College London, London, UK

**Keywords:** IPV, Intimate Partner Violence, IPV, HIV, Virtual, Communication, Online, Participation

## Abstract

There is increasing interest in the potential to deliver participatory dialogical HIV and intimate partner violence (IPV) prevention interventions via digital platforms, though the majority of mHealth interventions have been didactic in approach. We undertook 10 in-depth interviews with male Peer Navigators (PNs) who had been extensively trained and working on a larger intervention promoting young people's sexual and reproductive rights, in rural KwaZulu-Natal. Interviews focused on their, and their peers', use of technology in their everyday lives. Data were transcribed and translated, and subjected to thematic analysis. PNs described structural barriers to the use of technology, including poor connectivity, high data costs, and erratic electricity. They primarily used Facebook and WhatsApp for communication and highlighted how reading messages asynchronously was important to overcome connectivity challenges. PNs shared how groups were primarily for information sharing, they also discussed ‘sensitive’ issues online. Privacy was a concern, especially for conversations, and there was recognition of how confidentiality could be breached. It was also felt that WhatsApp could potentially support greater openness in discussions. We reflect on the potential for online interventions to support dialogical health communication, highlighting how dialogical health communication may be enabled through information provision, the asynchronous communication enhancing the potential for reflection, and greater participation in discussion by those who are shyer. Despite this potential there remain important risks around privacy of discussions and how to implement these approaches online.

## Highlights

• Qualitative research with young men showed high levels of cellphone usage.
• Emphasizes the importance of sharing information and personal discussions online.
• Suggests potential opportunities for discussion and reflection via WhatsApp.
• Recognises limitations of WhatsApp communication for health communication.

## 1 Background

HIV and intimate partner violence (IPV) are interlinked health and human rights issues, with bi-directional relationships (Dunkle and Decker, 2013; WHO, 2013). South Africa has the largest number of people living with HIV globally, with men, particularly young men, failing to engage in the HIV-treatment and HIV-prevention cascade (HSRC, 2018). Similarly, there are high rates of IPV perpetration by men in the general population, with one population-based study in South Africa reporting that 62 percent of men had perpetrated physical and/or sexual IPV at least once in their lifetime (Machisa et al., 2016).

These exceedingly high rates of HIV and IPV are intimately connected to South Africa's brutal history of apartheid, and apartheid's legacies of poverty, the destruction of family systems and the normalization of violence in everyday life (Seedat et al., 2009). These structural-historical factors are critical in shaping men's masculinities and impacting on young men's life chances, experiences in childhood and adolescence, of poverty, violence and gender inequalities, which mould both men's perpetration of violence, as well as their limited engagement in the HIV-prevention and -treatment cascades (Baisley et al., 2018; Colvin, 2019; Fleming and Dworkin, 2016; Gibbs et al., 2018).

Participatory, group-based interventions have been a mainstay of IPV-prevention and HIV-prevention and treatment adherence interventions (Kerr-Wilson et al., 2020; Spaan et al., 2020) driven by recognition that group-based approaches can reach more people and support behaviour change. These interventions are delivered by a facilitator in small and often single-sex groups and rely on intensive participatory methodologies (Campbell, 2003; Gibbs et al., 2015). Key components of participatory interventions have been the use of discussion to deliver scientifically correct information and to support participants to reflect on the challenges they face in life (Campbell and Jovchelovitch, 2000; Vaughan, 2011). These interlinked components (information, dialogue, and reflection) have been referred to in social psychological approaches as a dialogical approach to health communication, and contrast with didactic approaches of health promotion which focus only on information provision (Campbell and Jovchelovitch, 2000; Campbell and Scott, 2011).

mHealth approaches to promoting health have often focused on information transfer, or didactic health communication. Much work has focused on the use of Short Messaging Service (SMS) messages to support improved health outcomes. For instance, a 2016 review of mHealth interventions in low- and middle-income (LMIC) settings found that the majority of interventions relied on SMS messaging, often in one-way approaches (Hurt et al., 2016). Similarly, a 2017 review of HIV management interventions found that 16/41 relied on SMS messages to promote adherence, while a further 5/41 used applications to provide reminders and information on adherence (Cooper et al., 2017). Some interventions relied on calls to mobile phones and these included problem solving support (Cooper et al., 2017) and were often more interactive (Adeagbo et al., 2021). A review of online interventions for IPV-prevention found that these emphasized individual responses, with little consideration of contextual factors shaping women's responses (Rempel et al., 2019), while a different review on the same topic found online interventions were often focused on provision of information and safety planning (Anderson et al., 2019). Notably, all these IPV-prevention interventions have been focused on supporting women as survivors of violence with the majority of mHealth or online interventions typically narrowly focused on didactic approaches to health promotion, which also neglects the role of men as perpetrators of violence, and addressing the wider causes of IPV.

Alongside the rapid expansion of information and communications technology (ICT) in the global south, the COVID-19 pandemic and subsequent lockdowns has led to a consideration of the role of online methods to deliver HIV- and IPV-interventions. While there has been a growth in research on the delivery of participatory research online, this has primarily been in the global north (Hall et al., 2021; Nind et al., 2021). Given the rapid growth in technological reach in the global south, alongside the push to deliver interventions ‘online’ there, remains a lack of understanding of whether this can translate to the delivery of participatory interventions online and the potential opportunities and challenges of such approaches. There also remains almost no documented data on these approaches. As such, in this paper we seek to understand young (aged 18–35) male trained peer navigators' (Shahmanesh et al., 2021) and their peers' use of technology for communication, as well as their thoughts of delivering interventions online, in rural KwaZulu-Natal, South Africa.

## 2 Methods

### 2.1 Setting

The research was conducted in the Hlabisa sub-district of UMkhanyakude district in northern KwaZulu-Natal, South Africa, which is a mostly rural setting with some small but more dense communities and a large town to which people often travel. There are high levels of youth unemployment, with an estimated 85% of those aged 18–24 unemployed, and two-thirds of households receive social grants. The HIV-treatment cascade for young men (16–29 years) is also poor; in a recent trial, less than 80% knew their HIV-status, 25% were on treatment and 18% were virally suppressed (Iwuji et al., 2016, 2018). Access to mobile phones in 2017 was quite high, with 92 percent of those aged 18–35 years reporting mobile phone ownership, although less than half reported using social media (Shahmanesh et al., 2019).

### 2.2 Ethics

The study received ethical approval from the South African Medical Research Council's Human Research Ethics Committee, the Biomedical Research Committee of the University of KwaZulu-Natal, and the ethics committee of the University College London, UK. Participants provided verbal (recorded) informed consent for the study, prior to the interview commencing, because of the limitations imposed by COVID-19 on face-to-face interaction. Participants received no compensation for their time as they were paid a salary for their employment as Peer Navigators during this project, although they received airtime for related costs.

### 2.3 Participants and context

The study was conducted among young men who were hired as Peer Navigators as part of the African Health Research Institute (AHRI's) ongoing Thetha Nami peer navigator support intervention (Shahmanesh et al., 2021). Thetha Nami is a peer-led intervention to engage young people in HIV and sexual and reproductive health, specifically around increasing health system access through building bonding, bridging, and linking social capital in their communities. The Thetha Nami study team developed the intervention by recruiting and training young people (18–30 years) from the selected study communities. The intervention was co-created through a participatory process between the Thetha Nami team and young people, and this focused on addressing the challenges related to young people's access to HIV and sexual and reproductive health services. The final Thetha Nami intervention placed young people as professional area-based peer navigators who provided safe spaces and community advocacy and used a structured needs-assessment tool to tailor psychosocial support, peer mentorship and referral to appropriate health and social services in their local communities (Shahmanesh et al., 2021). The Peer Navigators have been working in the community since March 2019. During implementation of Thetha Nami, the Peer Navigators contacted over 40% of young people in the study community over a six-month period.

### 2.4 Data collection and analysis

We conducted one-on-one telephonic in-depth interviews with male Peer Navigators. The interviews were all remote, i.e. over the phone, because of COVID-19 regulations limiting physical interactions. Interviewers were trained researchers who spoke isiZulu fluently. Interviews took between 26 and 48 min, and were audio recorded. The interviews were then transcribed in isiZulu and then translated into English, and quality checked. All identifying information was removed.

Data were thematically analysed (Flick, 2002). Initially, the data were read and small codes were identified. These were then grouped together into meaningful sub-themes and then themes. The sub-themes and themes were discussed by the authorship team and revised in line with feedback and data. The findings presented here are the themes and sub-themes that were created from the data.

## 3 Results

In total, 10 Peer Navigators undertook an in-depth interview. They described using smartphones, though they highlighted how structural factors, including poor network connectivity and high data costs, limited their use of the internet. The Peer Navigators (PN) also described primarily using WhatsApp and Facebook to share information, but also how they shared more personal information online. PNs had lots of thoughts about the possibility of online interventions, including how its asynchronous nature could be helpful, as well as how the use of voice notes, rather than written messages, may improve communication. There was continued concern about the risks of sharing information online, but some PNs also highlighted how the relative anonymity of sharing online could help those who were shyer, and also enable people to speak who normally did not.

In all interviews, Peer Navigators described how almost all men in their communities had access to smartphones: “Yeah, let me say that in my community approximately 99 percent of men use smartphones.” (PN5) However, significantly fewer had access to laptops: “What can I say, some of them [men] have laptops and some don't. In fact, most don't have them.” (PN6). In terms of the apps men access via their smartphones, Peer Navigators generally spoke only about men using two main apps, WhatsApp and Facebook: “Yeah they have smartphones. They use them a lot. Especially for social networks like Facebook and WhatsApp.” (PN10). Other forms of apps were mentioned, including Twitter, Instagram, and betting apps, though these were much less commonly referred to.

While smartphones were common, not everyone had a smartphone, as one Peer Navigator commented: “Some guys have dumbphones here, but they [Facebook and WhatsApp] are not accessible, they are only accessible on smart phones.” (PN4). More widely smartphones were often old and had trouble working: “… sometimes you find that your phone isn't working well … it just has a few problems like the battery for instance.” (PN10).

All Peer Navigators described two sets of issues with smartphones as modes of communication: connectivity challenges and high data costs. In this relatively rural and remote area - though in reality experienced across much of South Africa, including in urban settings – men described how they often struggled with connectivity, especially for video calls:

“Yeah, network connection is usually problematic for video calls and WhatsApp calls. As you can hear from the call we are having, it's rare to have problems with this ordinary kind of call.” (PN7)

The challenges with network connections were partly linked to poor overall telecommunications network coverage, and these could be exacerbated by weather conditions and electricity outages:

“You want to communicate with other people perhaps through Facebook, when the power goes off the network also automatically goes off. So, it becomes difficult to communicate if there's no power.” (PN1)

“Where I am, the network connection is only a problem if the weather conditions are not good” (PN4)

High data costs were also a significant problem for the use of smartphones as a communication tool: “Another challenge is the affordability of data, not everyone can afford it. Smartphones constantly need updates.” (PN7).

Men did describe a range of strategies to try and overcome high data costs, including knowing where there were free Wi-Fi connections, which were often in shopping centres (malls) located in the nearest town, or having neighbours nearby willing to share their Wi-Fi connection. Other men described how they used apps which used less data:

PN10: Like Facebook has Free Mode that doesn't need you to have airtime to communicate with people.

Interviewer: Mhmm.

PN10: Yeah, if you have a small amount of data, you can also access WhatsApp you know. You don't need to go buy airtime and call someone. (PN10)

Others described how men would buy new SIM cards when these were offers from their mobile network provider offered along with free or cheaper data:

PN5: So, in most cases you used to bump into other guys in tuck shops there to buy data SIM cards; that SIM card used to cost R10, and it has 250MBs of data.

Men described how they primarily used the two apps, Facebook, and WhatsApp, on their smartphones for talking with friends: “I think it's a good means of relaxing and communicating” (PN6). The Peer Navigators described how they were often part of many groups where a variety of information was shared:

“On Facebook I'm in a group that keeps me up to date with the current affairs taking place in [local community] and another one about movies so I can stay abreast of the latest movies and series releases … And then in the group about job opportunities, everyone posts about job opportunities they may have seen so it enables me to apply to those I'm interested in.” (PN7)

An important aspect of WhatsApp for men's communication was that messages could be read asynchronously: “The benefit of WhatsApp groups is that one can reply to messages whenever they see them, regardless of what time it came through and what time you see it” (PN9). And similarly, PN8 said: “With WhatsApp, you can respond when it suits you.”

While group chats were generally seen positively, a number expressed concern with written messages. One issue was that some men may struggle to read (in either isiZulu or English) messages given the low levels of literacy among young men. A second challenge was when there were multiple written messages on a group chat and reading them was too much. One Peer Navigator suggested voice notes as a solution to this:

PN1: … getting perhaps 300 WhatsApp messages that I would hardly read because of not even knowing where to start reading them. Yet if there are voice notes I can just touch play and listen to that well: he said this, he said this, he said this. And then I can respond to something I understood clearly. It becomes difficult if you find lots of messages. So, perhaps if we could use voice notes, I could engage much better.

The Peer Navigators also discussed that groups could be more than simply information sharing spaces and could also be used to discuss personal issues:

PN2: Ah, other information we share used to be those chats about life in general around the area which has nothing to do with serious stuff. Perhaps we will discuss about girls or when do we go out together as guys …

Yet, a few Peer Navigators felt that maybe WhatsApp messages and group chats would not be perceived as ‘serious’ discussion: “Texting via WhatsApp or Facebook may dilute the seriousness of these conversations and there are already people that don't take AHRI's work seriously here. They will wonder: ‘this person says they want to talk to me about serious matters, yet they are texting me?’” (PN7).

We found a sophisticated digital literacy emerging around the challenges and opportunities of how to communicate online, as well as strategies to deal with this. Many of the Peer Navigators mentioned that people had limited concentration online. The maximum length of time that Peer Navigators felt that people could focus online ranged from 30 min to 2 h, though almost all felt that an hour or less was the correct time: “Perhaps 30–45 min to people is good because once it's above an hour, ay people can become bored” (PN4). As one Peer Navigator commented, any online conversation that was too long would lead to people disengaging:

PN8: Oh no, for starters it could be like 30 minutes.

Interviewer: Oh, that's how long our group discussions should last for starters?

PN:8 Yeah, because sometimes lots of information at once can be so overwhelming that one ends up not grasping anything. Some people even shut down and alienate themselves completely.

Peer Navigators were also able to think of strategies to overcome challenges of concentration: “People can engage for long periods over the phone as long as they find the conversation interesting” (PN7). The use of humour was also suggested: “We should crack a joke every now and then so that people can feel relaxed. It should feel like a conversation we are having face-to-face” (PN10).

Peer Navigators were also aware of the challenges of not being able to see people and how this impacted on reading body language:

“Okay yeah it may happen the way it may happen but once we've discussed for a bit longer perhaps you may end up talking alone, or else when there's nothing that will free or make them laugh during the discussion you may find yourself lonely, they may all disconnect.” (PN2)

Similarly, conveying tone was described as a challenge in written messages:

PN7: Sometimes you are shouting, and you are angry when you type, but it's hard to convey that via text.

Interviewer: [Laughs] and sometimes you're not shouting or angry but you're talking very politely.

PN7: But then it comes across as shouting to the person on the receiving end.

More widely, driven by their prior experiences of discussions online, some men were worried that groups could end up having arguments, particularly about challenging issues, and these were often hard to moderate and deal with online:

PN9: I think not being able to see one another face to face is a disadvantage as it can breed misinterpretation and confusion in some people. There are certain emotions that don't quite come across when communicating via social media as opposed to face-to-face.

Three interlinked issues of privacy, confidentiality and anonymity of online discussions and group chats were raised by Peer Navigators. Many of the men described how privacy in the ‘real world’ was shaped by whether or not a man had his own room – particularly an outside room he had built within their family plot of land: “Yes, men in most cases have their own backrooms. Even if there is the main house, guys don't usually stay in it or do his own things there. He has his own house [backroom]. So, that's where I think they also get their privacy” (PN1). It was clear though that not all young men had such a space and would therefore potentially struggle with privacy. Other participants would have no way of knowing that a person was not in a private space, and this could disrupt discussions:

PN2: Yeah, those are some of the things, to find that other guys sleep in the bedroom and his mother has full access to it perhaps she is the one who washes his clothes or anything like that. Then you find that the discussion is about something serious, and the mother will just pop in, and then find that you can't do anything after that.

The possibility of other persons being present and over-hearing a conversation concerned Peer Navigators because of the implications for ensuring confidentiality of private group discussions. Indeed, this was experienced by the research team during one of the interviews with a Peer Navigator. Despite the team checking verbally with the Peer Navigator that he was in a private space, it became evident that he was not, but in a setting with other people:

Interviewer: What's good and bad about using things like WhatsApp and Facebook to communicate.

PN8: Please say that again?

Interviewer: It sounds like there are people there in the background, so you won't hear me properly now.

PN8: I will be able to speak, we can continue.

Interviewer: Pardon.

PN8: I said I will manage. Let's continue.

While in the case of this interview, the background noise and other voices were not a significant challenge as nothing sensitive was discussed, for more sensitive discussions envisaged during online interventions, the possibility of others over-hearing conversations could concern all participants and inhibit discussion.

There were also concerns with how sensitive information could be shared by some participants with others outside the group, breaking confidentiality of online discussions:

PN10: Oh. Adverse outcomes include people revealing other people's secrets in groups, telling lies about others on Facebook and pretending to be someone you're not.

In contrast to concerns about privacy and confidentiality, several Peer Navigators suggested that the relative anonymity that the mobile phone provided enabled some people to have greater confidence to express themselves openly:

PN1: Yes, because I used to notice what I'm pointing out in my team's group that you find some people made their points in writing, but when they have to say something in the field face-to-face, no, he doesn't say anything. So, I just feel they become free when they are talking over his phone alone.

This was particularly the case with being able to type out messages, rather than contribute to an oral discussion: “Yeah, some people are afraid to speak, and they prefer to type out their problems.” (PN10).

Another Peer Navigator also suggested that using text to share problems in a group, rather than discussing them verbally, may be a good way to encourage people to share their own thoughts and issues, especially among a group of similar young men:

PN8: I prefer WhatsApp because one will be able to write out their problems.

Interviewer: Pardon?

PN8: We will be in a group, and everyone will be writing their problems, right?

Interviewer: Yeah.

PN8: There might not be a need for one to look … they can learn in the group because people aren't all the same, some of us can't open up and express ourselves. So, if we have a group in which we can do that as men, we might realise that our problems are similar.

## 4 Discussion

Among young men engaged in an ongoing HIV-prevention and treatment intervention as Peer Navigators supporting other young men's access to healthcare in rural KwaZulu-Natal, there was a strong emerging understanding around digital literacy, and the opportunities digital technologies could offer to support interventions: sharing information, enabling some inhibited men to share information, and providing opportunities to talk about important topics. Yet, the Peer Navigators' views and sense of opportunities were constrained by two sets of interlinked structural realities. The first was the very poor network and slow connectivity in the community they lived in, which was significantly worse than in other areas of South Africa because of hills and more limited base stations. This was exacerbated by a range of issues, including the high costs of data and electricity outages, further limiting their ability to engage with and use smartphones to their full capability, and reflects findings elsewhere (Adeagbo et al., 2019; Hall et al., 2021; Porter, 2012). The second limitation was that they tended to rely heavily on WhatsApp and Facebook as they were low data usage apps, therefore cheaper to use, and allowed asynchronous communication. This may also reflect the relatively limited ability men had to engage with and navigate new technologies. In this discussion, we reflect on opportunities for participatory interventions to leverage available technologies in the context of these constraints.

A clear finding was that virtual communication could support the sharing of information on many topics. Peer Navigators described how they, and other men, were widely engaged in sharing information on a range of issues including job opportunities and community issues. The role of cellphones in sharing information around livelihoods and job opportunities is something described previously (Porter, 2012). Many mHealth interventions to date have as their dominant approach used technology as a form of didactic sharing of information or promotion of certain health behaviours (Anderson et al., 2019; Cooper et al., 2017; Hurt et al., 2016). While studies have shown some successes, others have highlighted how structural constraints and challenges have undermined simple information sharing and promotion of health outcomes (Govender et al., 2019). Despite these limitations, the opportunity to share basic information remotely, supporting the wider aims of an intervention and promoting access to health care, remains an important one that existing participatory interventions can easily leverage.

There was also recognition by the Peer Navigators that it was possible to move beyond information sharing to discuss ‘sensitive’ issues online. Many of the men themselves described how they engaged in lively discussion and debate online, regarding topics including, but not limited to, relationships. They also noted that there were numerous challenges associated with this, ranging from connectivity and data issues through to others misreading tone and body language. They were also aware of the way online discussions did not necessarily reflect ‘real world’ discussion.

A key finding was that Peer Navigators thought discussions online through text messages or voice notes enabled a sense of greater anonymity and thus supported shyer participants to be more open in discussions of sensitive topics. This anonymity was different to that described in online chat forums in high-income settings, where people do not know each other and use pseudonyms, and were more comfortable discussing sensitive topics as compared to face-to-face conversations (Jones et al., 2011; Prescott et al., 2017). Rather, the anonymity described by the peer navigators on WhatsApp conversation was potentially related to not seeing other people's immediate responses to any comment made by a participant (Colom, 2021). The options of using either written messages or voice notes was also appreciated, as some felt it was easier to write out an issue rather than physically voice it, while others preferred the ease of simply listening to messages. Developing strategies of supporting online communication and discussion to supplement face-to-face activities of participatory interventions appears an important approach.

Despite the recognition that discussions of sensitive topics could occur online, Peer Navigators also recognised that simultaneous discussion was unlikely given connectivity challenges and the ways in which people engaged with mobile phones, and asynchronous communication was more common (Colom, 2021). Participatory interventions assume a real-time conversation, with everyone in the same physical or virtual space at the same time (Morrison-Smith and Ruiz, 2020). Yet, in the contexts these young men lived in, the virtual space was not simultaneous. Indeed, Colom (2021) suggests that it may be possible to reimagine how WhatsApp communication can support reflective strategies, even if communication is asynchronous. Developing ways of supporting asynchronous discussion may be important for online interventions.

Throughout interviews there were ongoing concerns about privacy and the risk of breaching confidentiality of discussions that were conducted online. One issue was whether other people could overhear what was being discussed by participants, although the issue was less of a concern for Peer Navigators who often had private spaces (rooms) to do discussions in. Another issue was the confidentiality of messages. Although not explicitly raised, prior research has highlighted how intimate partners often access the phone messages of each other as a way of checking for infidelity (Archambault, 2011; Gibbs et al., 2021), which has implications for sharing sensitive messages to a group via WhatsApp. Additionally, people in the group could share other people's messages outside the group; even with the delete function on WhatsApp, people can still ‘screenshot’ messages with identifying information. While this is an ongoing concern for any participatory intervention, sharing of text messages and voice notes provides written or audio evidence of what one person said and moves from ‘gossip’ to ‘fact’. This reflects Archambault (2011), who in Mozambique described how messages reflecting a partner's infidelity were more harmful than rumors as they were written proof of cheating, rather than simply something that could be dismissed as hearsay and strategically ignored. The risk for online messages, and voice notes, is that they too become currency in the sharing of information outside of groups.

This study has a number of limitations. We only interviewed a small, very selective group of young men about their use of technology and their perceptions of their peers’ use of technology. As such, this limits the generalizability of findings. However, the group of Peer Navigators had some understanding of participatory activities and interventions, through being involved in multiple group activities in their training, and were closely embedded in the community which they came from through their involvement in the Thetha Nami intervention. Expanding this research to other groups of young men would be an important step in improving reliability of the work. We also interviewed men, and it is not clear the extent to which young women had similar or different access to cellphone technology and data in this context, as previous studies have suggested that this is often shaped by gendered power relations (Gibbs et al., 2021). The area also had very poor internet connection, and therefore the results can only be considered representative of areas with similar experiences.

## 5 Conclusion

The potential for dialogical interventions to be delivered online, given the massive constraints for young men in rural South Africa in terms of poor network access, high costs and limited access to technology, suggests that simply transferring the methodology of face-to-face participatory interventions from physical to virtual spaces is unrealistic. This is in stark contrast to work and research in the global north, where participatory research and interventions are being developed and implemented (Hall et al., 2021; Jones et al., 2011; Nind et al., 2021). Despite the challenges for online interventions in rural South Africa, Peer Navigators had a sophisticated understanding of the opportunities and constraints presented using by using communications technologies and how these could be translated into online dialogical health communication.

This study suggests that there are potential areas in which dialogical health communication can be supported online. First, at a basic level, using technology to promote key messages and information supporting the objectives of an intervention, such as where to access health systems or look for jobs, supports basic didactic health communication. Second, there needs to be an expansion towards imaging what dialogical communication online could look like. WhatsApp communication, though asynchronous, can give greater time for reflection and promote quieter participants to engage more given the relative anonymity of online communication. This prompts a series of future research questions primarily around implementing online interventions. Questions include how to build trust and relationships online, and whether blended interventions, combining both physical and remote sessions work better than online only sessions. It also requires answering questions such as how best to create privacy in online discussions, and importantly, how to ensure dialogical communication – discussion and reflection – can be achieved. This will be best resolved through small pilots with extensive qualitative research.

## Funding

Funding for this project was received from the UK Medical Research Council (MR/T025832/1). Africa Health Research Institute is supported by a grant from the 10.13039/100010269Wellcome Trust (082384/Z/07/Z). MS is supported by the 10.13039/100000002National Institutes of Health under award number 5R01MH114560-03**.** Additional funding was from the 10.13039/501100001322South African Medical Research Council (YS). The funders had no input into study design, data collection, analysis or decision to publish.

## Credit author statement

Andrew Gibbs – conceptualization; Formal analysis; Funding acquisition; Methodology; roles/writing – original draft. Dumsani Gumede – data curation, Investigation, Project administration, Writing – review & editing. Manono Luthuli – data curation, Investigation, Project administration, Writing – review & editing. Zakhele Xulu – data curation, Investigation, Project administration, Writing – review & editing. Laura Washington – conceptualization, Writing – review & editing. Yandisa Sikweyiya–; conceptualization, Writing – review & editing. Oluwafemi Adeagbo – conceptualization, Writing – review & editing. Maryam Shahmanesh – conceptualization, Funding acquisition, Project administration, Writing – review & editing.
